# Codon-Dependent Transcriptional Changes in Response to Tryptophan Limitation in the Tryptophan Auxotrophic Pathogens Chlamydia trachomatis and Streptococcus pyogenes

**DOI:** 10.1128/mSystems.01269-21

**Published:** 2021-12-14

**Authors:** Scot P. Ouellette, Nathan D. Hatch, Nicholas A. Wood, Andrea L. Herrera, Michael S. Chaussee

**Affiliations:** a Department of Pathology and Microbiology, University of Nebraska Medical Center, Omaha, Nebraska, USA; b Division of Basic Biomedical Sciences, Sanford School of Medicine, University of South Dakota, Vermillion, South Dakota, USA; Leiden University

**Keywords:** *Chlamydia*, *Streptococcus*, stringent response, *relA*, tryptophan, persistence, interferon gamma

## Abstract

Chlamydia trachomatis and Streptococcus pyogenes are among the most prevalent bacterial pathogens of humans. Interestingly, both pathogens are tryptophan (Trp) auxotrophs and must acquire this essential amino acid from their environment. For Chlamydia, an obligate intracellular bacterium, this means scavenging Trp from the host cell in which they reside. For Streptococcus, a primarily extracellular bacterium, this means scavenging Trp from the local environment. In the course of a natural immune response, both pathogens can be exposed to Trp-limiting conditions through the action of the interferon gamma-inducible IDO1 enzyme, which catabolizes Trp to *N*-formylkynurenine. How these pathogens respond to Trp starvation is incompletely understood. However, we have previously demonstrated that genes enriched in Trp codons were preferentially transcribed in C. pneumoniae during Trp limitation. Chlamydia, but not Streptococcus, lacks a stringent response, which is a global regulon activated by uncharged tRNAs binding in the A site of the ribosome. We hypothesized that the chlamydial response to Trp limitation is a consequence of lacking a stringent response. To test this, we compared global transcription profiles of C. trachomatis to both wild-type and stringent response mutant strains of Streptococcus during Trp starvation. We observed that both Trp auxotrophs respond with codon-dependent changes in their transcriptional profiles that correlate with Trp codon content but not transcript stability. Importantly, the stringent response had no impact on these transcriptional changes, suggesting an evolutionarily conserved adaptation to Trp starvation. Therefore, we have revealed a novel response of Trp auxotrophic pathogens in response to Trp starvation.

**IMPORTANCE** Chlamydia trachomatis and Streptococcus pyogenes are important pathogens of humans. Interestingly, both are auxotrophic for tryptophan and acquire this essential amino acid from the host environment. However, part of the host defense against pathogens includes the degradation of tryptophan pools. Therefore, Chlamydia and Streptococcus are particularly susceptible to tryptophan starvation. Most model bacteria respond to amino acid starvation by using a global regulon called the stringent response. However, Chlamydia lacks a stringent response. Here, we investigated the chlamydial response to tryptophan starvation and compared it to both wild-type and stringent response mutant strains of S. pyogenes to determine what role a functional stringent response plays during tryptophan starvation in these pathogens. We determined that both of these pathogens respond to tryptophan starvation by increasing transcription of tryptophan codon-rich genes. This effect was not dependent on the stringent response and highlights a previously unrecognized and potentially evolutionarily conserved mechanism for surviving tryptophan starvation.

## INTRODUCTION

How organisms respond to stress is an area of intense study. This is particularly true in microbial pathogenesis as a bacterium’s ability to survive and adapt to stress has important ramifications for human health. For example, treatment with antibiotics can lead to phenotypic resistance in bacterial populations defined by so-called “persister” cells (1–3). Persistent cell populations of bacteria can also be induced in the face of a host’s immune response, whereby the pathogen can enter a dormant metabolic state that allows for long-term survival, such as occurs for Mycobacterium tuberculosis (4). A better understanding of how diverse bacteria respond to stress may reveal evolutionary adaptations that could ultimately be exploited for antimicrobial development or diagnostic applications.

Chlamydia and Streptococcus represent two evolutionarily distinct phyla of bacteria with very distinct ecologies and physiologies. Chlamydia trachomatis is an obligate intracellular, Gram-negative bacterium causing sexually transmitted diseases and trachoma, a blinding disease (5). Streptococcus pyogenes is a Gram-positive bacterium, primarily extracellular, associated with severe, often toxigenic, diseases in humans, but it can also be a resident of the host microflora without causing disease (6). Interestingly, both pathogens are auxotrophic for tryptophan (Trp) and acquire this essential amino acid from their host environment (7). This is important because, in responding to these pathogens, human immune cells can produce the cytokine interferon gamma (IFN-γ) (8). IFN-γ can activate human cells to produce the enzyme indoleamine-2,3-dioxygenase (IDO), which catabolizes Trp to the nonutilizable product *N*-formylkynurenine (8–10). Thus, one consequence of IFN-γ production by the immune response is the establishment of a Trp-limiting environment. For Trp auxotrophic organisms, this results in Trp starvation.

In response to amino acid starvation, many bacteria, including Streptococcus, induce the stringent response (11). When an uncharged tRNA binds in the A site of the ribosome, the ribosome-associated enzyme RelA is activated to produce (p)ppGpp. The production of (p)ppGpp has pleiomorphic effects and acts as a signaling molecule to alter transcription and other processes in the bacterial cell, which result in downregulation of translation and stable RNA synthesis (e.g., 16S rRNA) and activation of amino acid biosynthesis and proteolytic pathways that restore amino acid pools (12). Chlamydia lacks RelA and does not produce (p)ppGpp, yet is exposed to Trp starvation during IFN-γ activation of the host cell (13). Therefore, how Chlamydia responds to amino acid stress in the absence of a stringent response represents an intriguing microbiological question.

The morphologic effects of IFN-γ activation of human epithelial cells and subsequent IDO production on Chlamydia have been well characterized (8, 14–22). Chlamydia trachomatis cycles between two developmental forms during an infection (5). The elementary body (EB) is the nonreplicative infectious form, while the reticulate body (RB) is the noninfectious yet replicative form. After uptake into the cell, the EB differentiates to the RB within a membrane-bound vacuole (termed an inclusion), which rapidly diverges from the endocytic pathway (23). The RB continues to develop and replicate within the inclusion, asynchronously differentiating into EBs until the host cell lyses or the inclusion is extruded. When starved for Trp by IDO activity, the chlamydial developmental cycle is diverted into “persistence,” which is characterized by nondividing, aberrantly enlarged RB forms (8, 18–21). Importantly, chlamydial persistence is reversible, such that upon repletion of Trp, Chlamydia reverts back from the persistent state to the developmentally competent RB state, resuming the developmental cycle (17, 19, 22). Thus, chlamydial persistence represents a means by which the pathogen resists a key immune response.

The exact molecular mechanisms involved in entering, maintaining, and exiting persistence are poorly understood. A common assumption is that Chlamydia responds in a specific manner by enacting a global regulon. However, we have previously challenged this by demonstrating a disconnect between transcription and translation (17). More recently, we identified a correlation between genes encoding Trp codons and increased transcription during persistence (22). Conversely, transcription of genes that do not encode Trp codons either decreased or did not change during persistence. This effect was not related to decreased degradation of Trp codon-rich transcripts, as we monitored transcript half-life during persistence as a function of Trp codon content and observed no differences between Trp codon-rich or Trp codon-poor transcripts (22, 24). Therefore, we hypothesize that Chlamydia responds to Trp starvation in a Trp codon-dependent manner. This novel, albeit counterintuitive, response to the depletion of an essential amino acid led us to further hypothesize that Chlamydia may employ a “primitive” adaptation to survive starvation that, in other Trp auxotrophs, has become obsolete by the evolution of the stringent response.

To further investigate this hypothesis, we first performed RNA sequencing (RNA-seq) on C. trachomatis in the presence and absence of either Trp or leucine (Leu), using characterized bacterial tRNA synthetase inhibitors we recently demonstrated induce persistence in Chlamydia (25). IFN-γ-treated cultures were also included to compare IDO-mediated Trp starvation. To determine the role of a functional stringent response in transcriptional changes elicited by Trp starvation in a Trp auxotroph, we performed RNA-seq on S. pyogenes wild-type or *relA* mutant strains during Trp starvation. We subsequently analyzed all transcriptional changes based on amino acid codon content of the transcript. We determined that both species respond to Trp starvation by alterations in transcript levels that correlate with the Trp codon percentage. Interestingly, this effect was independent of RelA. Furthermore, the increase in Trp codon-containing transcripts was not due to increased protection of these transcripts from ribosome stalling. Half-life measurements of transcripts from Streptococcus during normal conditions and Trp starvation revealed a general increase in transcript stability that was not correlated with Trp codon content. Our findings suggest an evolutionarily conserved adaptation to Trp starvation not previously described.

## RESULTS

### Amino acid limitation mediated by IFN-γ activation of host cells or by inhibitors of bacterial tRNA synthetases induces persistence in Chlamydia.

**FIG 1:**
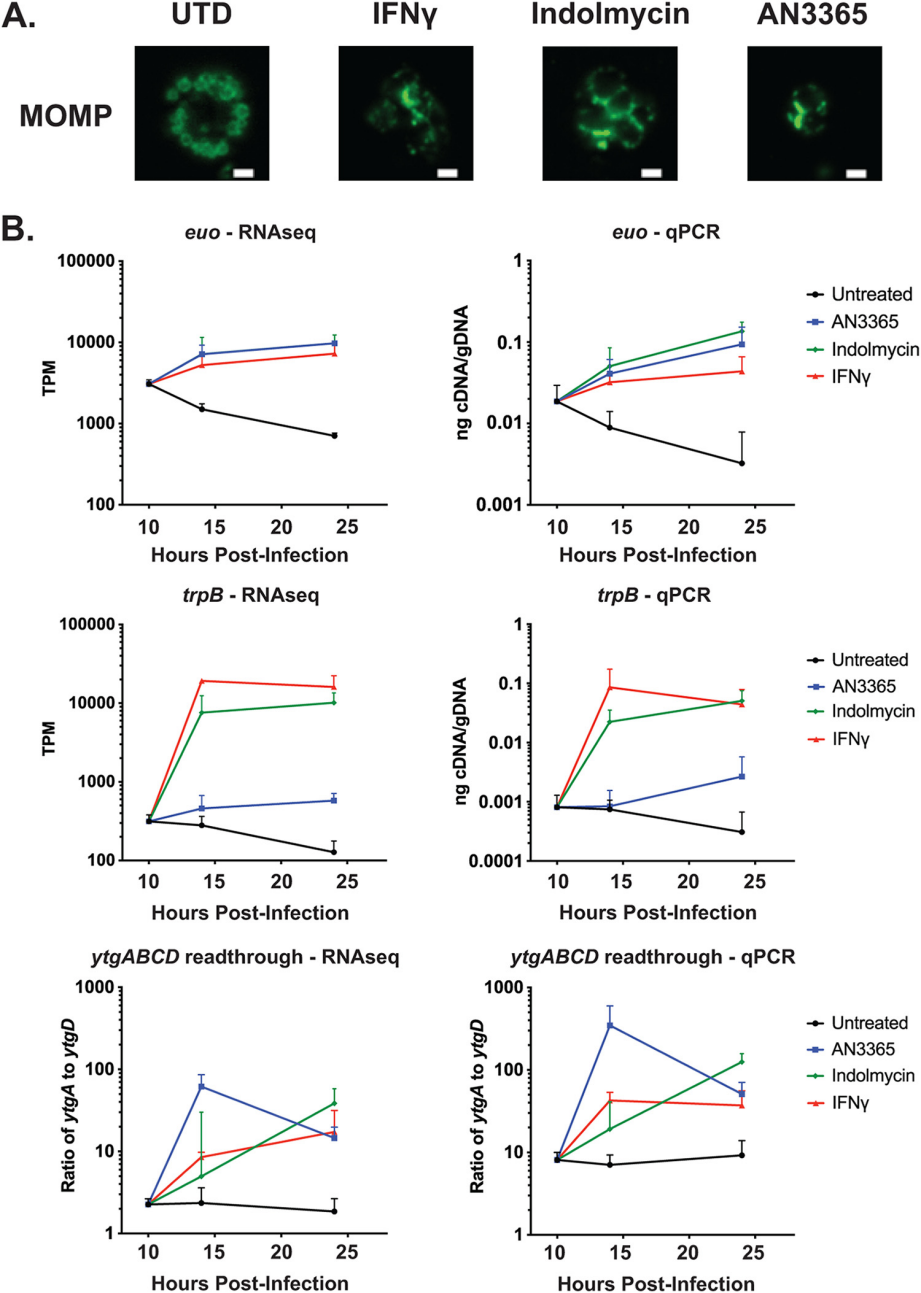
Each model of chlamydial persistence results in expected morphological and transcript phenotypes. (A) Representative images of HEp-2 cells infected with C. trachomatis and treated or not as described in Materials and Methods. Cells were fixed with methanol and stained 24 hpi using primary antibodies to major outer membrane protein (MOMP). Indolmycin and AN3365 treatments resulted in smaller inclusions and morphologically aberrant organisms, similar to IFN-γ-treated organisms. All images were acquired on an Axio Imager.Z2 with ApoTome.2 at ×100 magnification. Scale bars represent 1 μm. (B) To determine whether our RNA-seq purification steps grossly altered sample integrity, TPM (transcripts per million) values of *euo* and *trpB* (left) were compared to RT-qPCR data of the same biological replicates prior to RNA enrichment protocols (right). Additionally, TPM of *ytgA* and *ytgD* were used to calculate readthrough efficiency and compared to an RT-qPCR equivalent. RT-qPCR graphs (right) are modified from reference 25.

We recently validated the use of bacterial tRNA synthetase inhibitors indolmycin and AN3365 as useful tools to induce persistence in Chlamydia in an amino-acid-dependent manner (25). Due to the wide range of effects on host cells during IFN-γ exposure (26), we included these tRNA synthetase inhibitors to reduce the number of potential variables unrelated to amino acid limitation. Furthermore, while indolmycin targets the tryptophanyl-tRNA synthetase, creating a Trp-limited environment similar to IFN-γ, AN3365 targets the leucyl-tRNA synthetase (27–29). Inducing persistence by limiting an amino acid other than Trp will allow us to distinguish between effects specific to Trp limitation and broad effects of amino acid limitation since Leu is present in all but two proteins. As shown in Fig. 1A, each of these treatments can induce changes in bacterial morphology characteristic of persistence.

### RNA processing does not impact transcriptional patterns.

As Chlamydia trachomatis is an obligate intracellular bacterium, the vast majority of isolated RNA from cultures is host derived. To reduce this background and to increase the potential read depth of our RNA sequencing experiments, we performed multiple purification steps to enrich the prokaryotic mRNA but, nevertheless, had only a small fraction of the total reads map to the chlamydial genome, particularly for early time points and persistent samples with the lowest overall biomass (Table 1). To ensure that transcriptional patterns we observed by RNA-seq were broadly consistent with our reverse transcription-quantitative PCR (RT-qPCR) analyses from total RNA extractions (i.e., before purification), we plotted RT-qPCR data normalized on a per genome basis in comparison to transcripts per million (TPM) from the RNA-seq data set. The samples used in this study were different technical replicates but the same biological samples used in our previous work, and we included the RT-qPCR data from reference 25 in comparison to our analysis using TPM data (Fig. 1B). Following removal of poly(A)-tagged mRNA and eukaryotic and prokaryotic rRNA, the trends between transcript levels measured were unchanged, and this included the relative ratio between the 5′ and 3′ ends of the *ytg* operon, as we previously described (22, 24, 25). Therefore, it is unlikely that the purification steps we employed broadly altered the transcript pool.

**TABLE 1:** Summary of RNA-seq read data for C. trachomatis experiments^*a*^

Parameter	Result for:								
	10 hpi UTD	14 hpi				24 hpi			
		UTD	IFN-γ	Ind	AN3365	UTD	IFN-γ	Ind	AN3365
No. of reads (millions)	132	123	115	123	124	130	127	118	133
No. of reads mapped to Chlamydia	114,351	350,254	290,146	237,395	231,965	4,477,471	77,088	785,085	344,399
% reads mapped to Chlamydia	0.09	0.28	0.25	0.19	0.16	3.42	0.06	0.66	0.25
% C. trachomatis reads − protein coding	93.78	96.20	98.89	96.09	96.39	97.92	92.31	97.76	96.51
% C. trachomatis reads – rRNA	4.58	2.00	1.08	1.13	2.13	0.47	4.43	0.63	2.12
Normalized fold genome coverage	7.75	24.20	6.90	10.96	16.07	316.42	5.09	55.36	23.87

a Shown is a compilation of data from CLC Genomics RNA-seq summary reports. UTD, untreated; Ind, indolmycin. Values are averages of all biological replicates. Normalized fold genome coverage was calculated by multiplying reads mapped to Chlamydia by 75 (bp per read), multiplying by % C. trachomatis reads − protein coding, and dividing by bp in the genome (1,038,842).

### A quintile analysis reveals patterns of transcriptional changes associated with the amino acid content of the encoded protein.

Most studies assessing transcriptional changes associated with persistence in Chlamydia typically compare an untreated control sample to the persistent sample to infer patterns of gene expression associated with persistence (30–32). As we have demonstrated in several studies, this type of analysis is flawed because it most often compares a persistent sample to one that transcriptionally resembles RBs differentiating to EBs (i.e., one in which late gene transcription has been activated) (17, 22, 24, 25). As persistent forms arise from RBs, a more pertinent analysis is to compare a persistent sample to the RB and to monitor transcriptional changes over time as persistence is established (i.e., a longitudinal analysis) (17, 22, 24, 25). To this end, we collected RNA from three time points: 10 h postinfection (hpi), 14 hpi, and 24 hpi. At 10 hpi for C. trachomatis L2, the EB has differentiated into the RB, and the RB is beginning its first division (33). At 14 hpi, peak RB-associated transcription is occurring (e.g., see reference 34). At 24 hpi, RBs have begun to differentiate to EBs, with late gene transcription increasing (35, 36). We induced amino acid starvation with the bacterial tRNA synthetases at 10 hpi: thus, the 14- and 24-hpi samples represent 4 h and 14 h of starvation, respectively. For IFN-γ-mediated persistence, we pretreated cells and exchanged the medium at 10 hpi for IFN-γ-conditioned medium (ICM), as described previously (25). To perform our analyses, we compared samples from 14 hpi to 10 hpi to assess early changes, 24 hpi to 14 hpi to assess late changes, and 24 hpi to 10 hpi to assess overall changes in transcription associated with persistence.

To gauge whether the amino acid codon content of a transcript impacts transcript levels during amino acid starvation, we analyzed our RNA-seq data for fold changes between the time points, as described above, as a percentage of the amino acid content of the encoded protein. The proteome averages for each amino acid and protein length, as well as the overall percentage of GC content (%GC) for Chlamydia trachomatis are shown in Table 2 (37). Note, the average gene size is approximately 1,050 bp. As the fold coverage of the RNA-seq reads for Chlamydia is much less than would be expected for a pure bacterial culture, we hypothesized that trends in transcriptional changes could be missed by only relying on a statistically significant data set. For example, we observed some transcripts were often changed more than 2-fold but had significance values between 0.05 and 0.1, suggesting there could be more variability due to the low biomass of Chlamydia in persistent cultures. We thus decided to rank order the fold changes from most upregulated to most downregulated for each comparison as an initial means to assess the data set. The data were analyzed by averaging each quintile (∼175 genes per quintile) for each amino acid as a percentage of the change in amino acid codon content compared to the proteome average (see Table S1 in the supplemental material). The average fold change in transcription for each quintile for each comparison is shown in Table 3. We also analyzed the data based on gene length, as we previously observed a positive correlation between increased fold changes between transcripts and smaller gene size during IFN-γ-mediated persistence.

**TABLE 2:** Proteome averages of each amino acid, average protein length, and %GC content for C. trachomatis L2 and S. pyogenes NZ131

Amino acid	Result for:	
	C. trachomatis L2	S. pyogenes NZ131 (GAS)
Proteome avg (%)		
Phenylalanine (F)	4.83	4.40
Leucine (L)	11.32	10.35
Isoleucine (I)	6.58	7.50
Valine (V)	6.68	6.74
Serine (S)	7.99	6.11
Proline (P)	4.26	3.21
Threonine (T)	4.89	5.74
Alanine (A)	7.55	7.53
Tyrosine (Y)	2.93	3.71
Histidine (H)	2.25	2.06
Glutamine (Q)	4.16	4.21
Asparagine (N)	3.33	4.31
Lysine (K)	6.12	7.42
Aspartate (D)	4.37	5.57
Glutamate (E)	6.57	6.52
Cysteine (C)	1.76	0.74
Arginine (R)	5.10	4.16
Glycine (G)	6.24	6.24
Methionine (M)	2.13	2.66
Tryptophan (W)	0.94	0.83

Avg. protein length (aa)	353	301

%GC	41.5	40.1

**TABLE 3:** Summary of the average fold change in transcript level within each quintile of the C. trachomatis RNA-seq data set^*a*^

Parameter	Fold change in transcript level (avg *P* value)				
	Quintile 1	Quintile 2	Quintile 3	Quintile 4	Quintile 5
14IFN/10U	3.791 (*P* = 0.12)	1.355 (*P* = 0.45)	−0.234 (*P* = 0.84)	−1.473 (*P* = 0.42)	−3.649 (*P* = 0.14)
14Ind/10U	2.749 (*P* = 0.18)	1.301 (*P* = 0.57)	0.133 (*P* = 0.89)	−1.259 (*P* = 0.61)	−2.130 (*P* = 0.27)
14AN/10U	3.567 (*P* = 0.07)	1.492 (*P* = 0.34)	−0.068 (*P* = 0.79)	−1.524 (*P* = 0.38)	−3.486 (*P* = 0.08)

24IFN/10U	3.276 (*P* = 0.12)	1.280 (*P* = 0.52)	−0.803 (*P* = 0.76)	−1.651 (*P* = 0.26)	−4.070 (*P* = 0.10)
24Ind/10U	4.385 (*P* = 0.06)	1.493 (*P* = 0.34)	−0.025 (*P* = 0.78)	−1.683 (*P* = 0.29)	−4.569 (*P* = 0.04)
24AN/10U	3.304 (*P* = 0.08)	1.482 (*P* = 0.36)	0.067 (*P* = 0.82)	−1.517 (*P* = 0.38)	−3.744 (*P* = 0.08)

a IFN, interferon gamma; Ind, indolmycin; AN, AN3365; U, untreated. 14/10 and 24/10 refer to the comparison at a particular time point (i.e., 14 or 24 hpi compared to 10 hpi). See also Fig. 2.

10.1128/mSystems.01269-21.2 TABLE S1 Quintile data from RNA-seq analysis of C. trachomatis serovar L2 grown under normal and amino acid starvation conditions. See also Fig. 2. Download Table S1, XLSX file, 0.2 MB. Copyright © 2021 Ouellette et al. 2021 Ouellette et al. https://creativecommons.org/licenses/by/4.0/ This content is distributed under the terms of the Creative Commons Attribution 4.0 International license.

A summary of the quintile analysis for the uppermost (upregulated) and bottommost (downregulated) quintiles is presented in Venn diagram form in Fig. 2. In general, we used a 10% change from the proteome average as a cutoff for biological relevance. This analysis revealed that, in the early phase of the response to amino acid limitation, transcripts smaller than the genome average (<900 bp) are disproportionately upregulated, irrespective of the starvation condition (“Smaller” in Fig. 2). IFN-γ-mediated tryptophan starvation resulted in upregulation of transcripts that were enriched in Trp codons (up arrow with W in Fig. 2). Both indolmycin and AN3365 resulted in upregulated transcripts with fewer (down arrow) Pro and His codons, whereas transcripts upregulated during AN3365 treatment were also enriched in Val and Cys codons. In contrast, transcripts larger than the genome average are disproportionately downregulated and also lower in Val and Gly codon content in the early phase. IFN-γ-mediated tryptophan starvation resulted in downregulation of transcripts that were enriched in His codons, whereas indolmycin treatment resulted in downregulation of transcripts that were poor in Trp codons. Both indolmycin and AN3365 resulted in downregulated transcripts enriched in Thr and Pro codons. Transcripts downregulated during AN3365 treatment were also poorer in Arg and Cys codons. Given the limited changes in amino acid codon utilization occurring during the late phase of the response during IFN-γ or AN3365 treatment, we did not diagram this.

**FIG 2:**
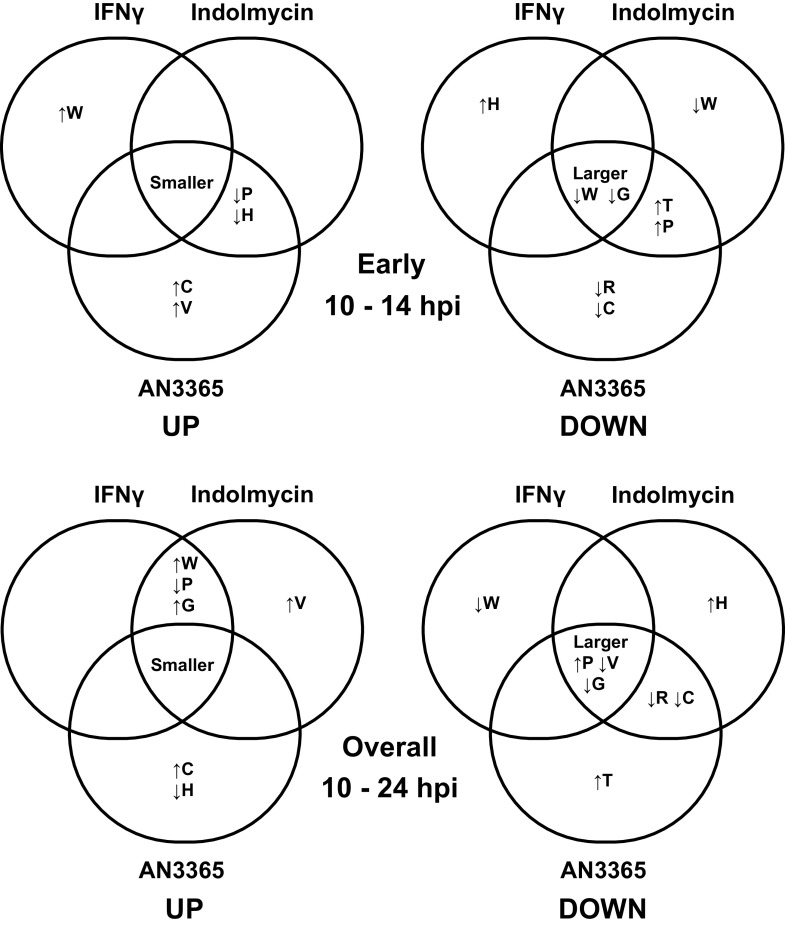
Quintile analysis of RNA-seq data during amino acid starvation-mediated persistence in Chlamydia trachomatis serovar L2. RNA-seq data sets were rank ordered from most upregulated to most downregulated for each comparison (e.g., 24-h AN3365 versus 10-h untreated). Each quintile of genes (∼175 ORFs per quintile) was averaged for its fold change (shown in Table 3) and amino acid content. Each comparison for both the upregulated (UP) and downregulated (DOWN) genes (i.e., the highest [quintile 1] and lowest [quintile 5] gene sets shown in Table 3) was then compared using a Venn diagram as shown. On top are shown early changes occurring during persistence between 10 and 14 hpi. On the bottom are shown overall changes occurring during persistence from 10 to 24 hpi. Up and down arrows next to amino acid code indicate a >10% increase or decrease, respectively, in the average percentage of that amino acid compared to the proteome average in the sample subset. “Smaller” and “Larger” indicate the average gene size within the sample subset is >10% smaller or larger, respectively, than the genome average (∼1,050 bp).

However, for the overall response, more similarities became apparent for IFN-γ or indolmycin treatment, suggesting that the latter is less efficient at inducing a Trp-limiting environment. This was expected based on our earlier observations and the fact that IFN-γ elicits enzyme-mediated Trp degradation via the action of IDO (25). For both Trp starvation conditions, upregulated transcripts were enriched in Trp and Gly and poor in Pro codons, with the largest magnitude of change being the Trp codon effect during IFN-γ-mediated Trp starvation. All starvation conditions showed upregulated transcripts of smaller genes; for each, downregulated transcripts of larger genes were also enriched in Pro and poor in Val and Gly codons. Overall, these findings are consistent with our prior results in Chlamydia pneumoniae, where we observed that upregulated transcripts during IFN-γ-mediated Trp starvation were enriched in Trp codons and smaller than the average gene length (22). Importantly, the Trp effects were specific to Trp starvation, as AN3365 did not elicit this effect. Finally, these data also suggest that translational stalling induced by limiting pools of charged tRNAs negatively affects the transcription of large genes.

### Codon-dependent transcriptional changes during amino acid starvation in Chlamydia.

**FIG 3:**
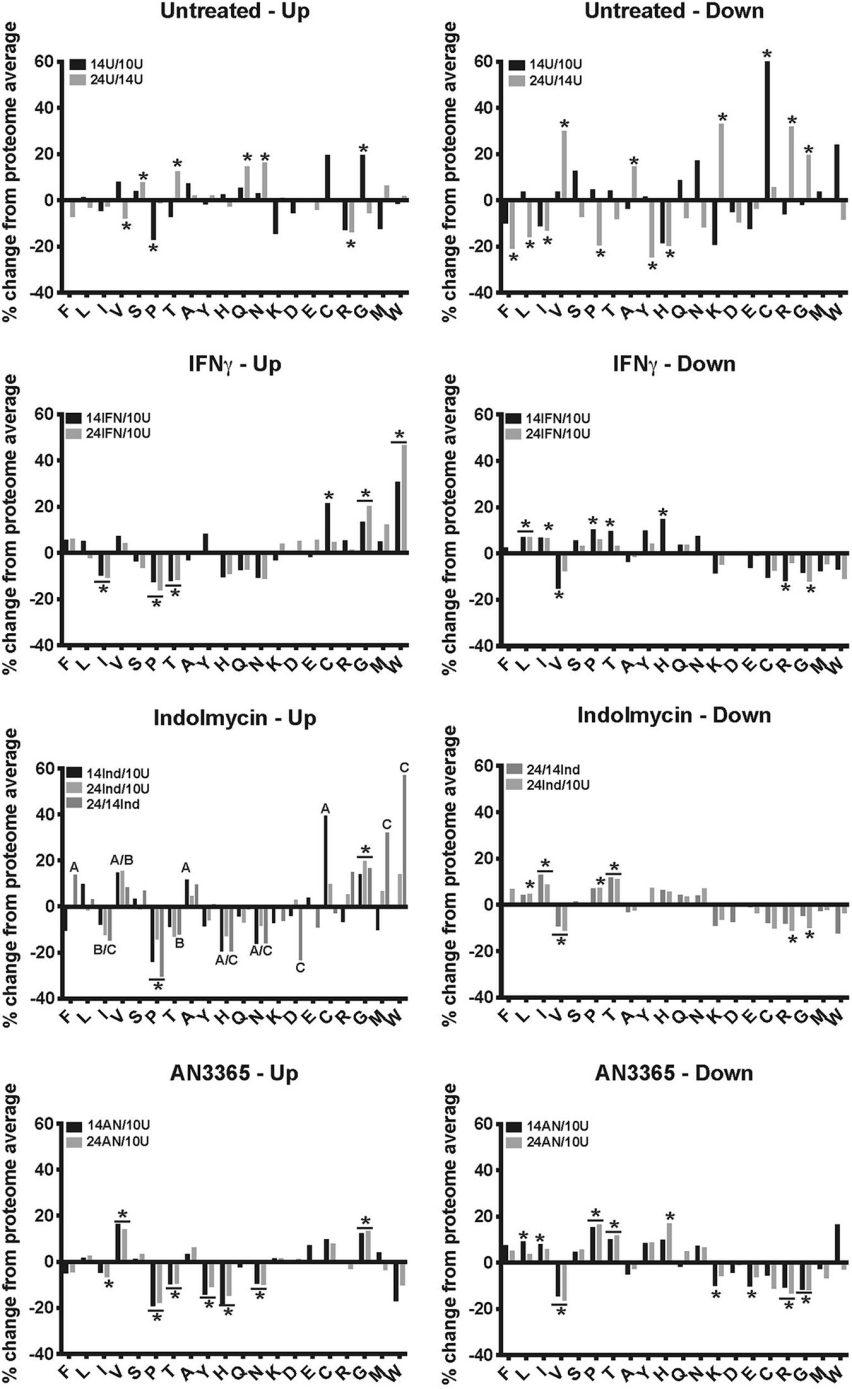
Codon content of genes differentially transcribed during chlamydial persistence deviates from the proteome average. Significant transcripts were defined as >2-fold changed, with a *P* value of <0.05. Significant transcripts were pooled for each sample based on up- or downregulation and graphed in accordance with the relative percentage of change in amino acid codon content compared to the proteome average. A two-tailed, unpaired Student's *t* test assuming equal variance was used to compare each subset of transcripts’ codon content to the entire gene set. * or * indicates the amino acid is significantly different (*P* < 0.05) from the proteome average for the indicated conditions. A/B/C indicate a *P* value of <0.05 for the indicated amino acid in the sample groups 14 hpi during indolmycin treatment versus untreated at 10 hpi (14Ind/10U), 24 hpi during indolmycin treatment versus untreated at 10 hpi (24Ind/10U), and 24 hpi versus 14 hpi during indolmycin treatment (24/14Ind), respectively. The number of genes per condition is further detailed in Table 4.

We next performed a stringent analysis of the RNA-seq data to determine whether the amino acid codon-associated changes in transcript levels were maintained with these smaller, statistically significant gene sets. To this end, rather than using a 1.5-fold cutoff, we analyzed 2-fold changes in transcript levels with significance values of *P* < 0.05. The total number of genes within each data set and the average gene length are presented in Table 4, and the top 15 most upregulated or downregulated genes for each condition are presented in Table S2 in the supplemental material. We excluded from further analysis comparisons for which 10 or fewer genes showed significant changes. This included the up- and downregulated genes in the 24-hpi versus 14-hpi (24/14h) IFN-γ data and the downregulated genes in the 14/10h indolmycin data. Of note, there were no statistically significant changes for the 24/14h AN3365 data sets, suggesting that this inhibitor induces a rapid and uniform starvation state similar to the enzyme-mediated Trp starvation state induced by IFN-γ. We then calculated the average gene length and amino acid codon content of the transcripts within each data set and graphed these averages as a percentage of change compared to the proteome average (Fig. 3). Significant changes from the proteome average are marked with an asterisk in the graphs.

**TABLE 4:** Summary of significant C. trachomatis transcripts and their relative length in comparison to the genome average^*a*^

Expression	Sample	No. of significant transcripts	Avg length (bp)	% change in length vs genome avg
Upregulated	14U/10U	30	1,211	14.38
	24U/14U	142	1,214	14.68
	14IFN/10U	54	847	−20.03
	24/14IFN	10	1,239	16.98
	24IFN/10U	61	1,003	−4.70
	14Ind/10U	39	861	−18.66
	24/14Ind	22	929	−12.27
	24Ind/10U	126	930	−12.14
	14AN/10U	103	871	−17.70
	24/14AN	42	1,112	4.96
	24AN/10U	100	896	−15.39

Downregulated	14U/10U	19	1,093	3.20
	24U/14U	51	655	−38.19
	14IFN/10U	75	1,480	39.78
	24/14IFN	9	818	−22.72
	24IFN/10U	105	1,306	26.90
	14Ind/10U	6	1,852	74.89
	24/14Ind	70	1,323	24.91
	24Ind/10U	141	1,430	35.02
	14AN/10U	93	1,297	22.44
	24/14AN	30	1,174	10.82
	24AN/10U	98	1,461	37.99

a Significant transcripts were defined as >2-fold changed, with a *P* value of <0.05. IFN, interferon gamma; Ind, indolmycin; AN, AN3365; and U, untreated. 14/10, 24/14, and 24/10 refer to the comparison at a particular time point (i.e., 14 or 24 hpi compared to 10 or 14 hpi). See also Fig. 3.

10.1128/mSystems.01269-21.3 TABLE S2 List of most increased (UP) and most decreased (DOWN) transcripts as determined from RNA-seq analysis of C. trachomatis serovar L2 grown under normal and amino acid starvation conditions. Download Table S2, XLSX file, 0.01 MB. Copyright © 2021 Ouellette et al. 2021 Ouellette et al. https://creativecommons.org/licenses/by/4.0/ This content is distributed under the terms of the Creative Commons Attribution 4.0 International license.

We first assessed amino acid changes associated with progression through the developmental cycle by comparing samples at 14 hpi versus 10 hpi and 24 hpi versus 14 hpi in untreated cultures. Not surprisingly, most amino acids showed no significant changes in their representation from the proteome average in the 14-hpi versus 10-hpi data set. As these are both primarily RB-centric phases of the developmental cycle, this is not surprising. However, significant differences in Pro (−17.1%) and Gly (+19.6%) codon content were detected. For downregulated transcripts at 14 hpi, only Cys (+66.4%) was significantly different from the 10-hpi sample. By 24 hpi, we observed many interesting changes in amino acid codon content and average gene size. For the upregulated data set, significant changes were observed in gene size (+14.7%) and Val (−7.9%), Ser (+7.6%), Thr (+12.5%), Gln (+14.7%), Asn (+16.3%), and Arg (−13.8%) codon content. Downregulated transcripts were significantly smaller (−38.2%), lower in Phe (−20.9%), Leu (−15.9%), Ile (−13.1%), Pro (−19.4%), Tyr (−24.7%), and His (−19.7%), and higher in Val (+30%), Ala (+14.5%), Lys (+33.2%), Arg (+31.8%), and Gly (+19.6%) codon content. These results may indicate that amino acid utilization by chlamydiae changes over the developmental cycle and may have important ramifications for developing axenic medium formulations that allow for transitions between developmental forms.

For simplicity, we describe the overall changes (24 hpi versus 10 hpi) in transcript patterns for the persistent samples. In general, genes upregulated during all persistence conditions in this comparison contained significantly fewer Ile, Pro, and Thr codons and more Gly codons. Both indolmycin and AN3365 treatments led to transcripts with more Val codons and fewer His and Asn codons. Similar trends were observed for IFN-γ treatment, but were not significant. All of these changes suggest general effects related to amino acid starvation. As noted in our quintile analysis, upregulated genes were significantly smaller for the IFN-γ-treated (−20%; *P* < 0.05) samples at 14 hpi, but this effect was not significant when comparing the 24-hpi versus 10-hpi conditions. Upregulated genes during indolmycin treatment showed a trend toward smaller sizes (−12.4% for 24 versus 10 hpi; *P* < 0.07), whereas upregulated genes during AN3365 treatment were significantly smaller (−15.4% for 24 versus 10 hpi; *P* < 0.05).

Importantly, upregulated genes in IFN-γ-treated samples exhibited higher Trp codon content, which displayed the highest change in codon usage of any amino acid (+47.1%; *P* < 0.00005). This Trp codon effect in indolmycin-treated samples was only significant when comparing the 14-hpi to 10-hpi samples (+57.1%; *P* < 0.005), but the trend was maintained when comparing 24-hpi to 10-hpi samples (+14.1%; *P* < 0.1). This was not the case for AN3365 treatment, as Trp codon content was reduced, albeit not significantly, in the upregulated gene set, highlighting the specificity of the increased Trp codon effect to Trp starvation states.

Conversely, downregulated genes contained significantly fewer Gly and Val codons across all treatments. IFN-γ or indolmycin treatment led to downregulated transcripts with more Ile and Leu codons, with AN3365 treatment displaying a similar trend. Again, these changes suggest general effects related to amino acid starvation. Indolmycin and AN3365 treatments resulted in downregulated transcripts with more Pro and Thr and fewer Arg codons. All persistent samples displayed downregulated transcripts from genes that were significantly larger (>20%) than the genome average.

One potential explanation for the increase in Trp codon-rich transcripts during Trp starvation is that ribosome stalling on these transcripts leads to an increase in their stability. We previously examined this in C. pneumoniae by measuring the half-life of selected transcripts that were rich or poor in Trp codons. We did measure a generalized increase in transcript stability; however, there was no Trp codon dependence to this (24). To determine whether this also applies to C. trachomatis in our current analysis, we measured the half-life of selected transcripts rich or poor in Trp codons by treating normal and persistently infected cultures with rifampin for 15 min to block RNA polymerase activity. Total RNA and DNA were collected and processed, and then transcript levels were measured. As with C. pneumoniae, we observed a general increase in transcript stability (Table 5). However, similarly to C. pneumoniae, the half-life of C. trachomatis transcripts during Trp starvation did not correlate with Trp codon content.

**TABLE 5:** C. trachomatis mRNA half-life is not correlated with Trp codon content^*a*^

Gene	C. trachomatis mRNA half-life (min) for:				#W^*b*^
	Untreated	AN3365	Indolmycin	IFN-γ	
*euo*	5.2	7.2	9.5	6.2	2
*groEL_1*	3.3	14.7	8.1	10.5	0
ytgA*	2.0	6.0	7.5	3.3	4
ytgD*	2.0	17.2	10.3	15.3	0
*rnhB_1^*	3.0	12.8	14.0	10.0	0
*metG^*	2.8	26.5	21.1	22.4	8
*mreB*	2.1	7.5	6.3	4.2	0
*Rho*	2.2	7.7	4.7	4.4	0
*clpP_2*	2.1	9.6	5.6	3.5	0
*omcB*	3.8	22.5	13.8	7.9	5

a RNA was collected at 14 hpi (Untreated) or 24 hpi (AN3365, indolmycin, or IFN-γ), with or without 15 min of rifampin (Rif) treatment, and quantified via RT-qPCR. mRNA half-life (*t*_1/2_) was calculated using the formula *t*_1/2_ = *t*/{log_2_(*N*_0_) − log_2_[*N*(*t*)]}, where *t* represents the 15 min of treatment with Rif, *N*_0_ is the amount of transcript measured at the time of addition of Rif, and *N*(*t*) is the amount of transcript measured after 15 min of Rif treatment. Data are expressed in minutes. * or ^ indicates contained within the same operon.

b #W, Trp codon content.

Overall, our analyses demonstrate two principal findings: (i) the specificity of increased Trp codon content of upregulated transcripts during Trp starvation and (ii) the negative impact of amino acid starvation on larger transcripts. Additionally, as we previously observed for C. pneumoniae, the increase in Trp codon-containing transcripts cannot be attributed solely to an increase in their stability. Importantly, as with our prior microarray analysis in C. pneumoniae (22), none of these analyses considers the impact of amino acid starvation effects on operons, which may potentially mask even more dramatic impacts on codon content.

### Large or Trp codon-rich transcripts or operons are incompletely transcribed during Trp starvation.

**FIG 4:**
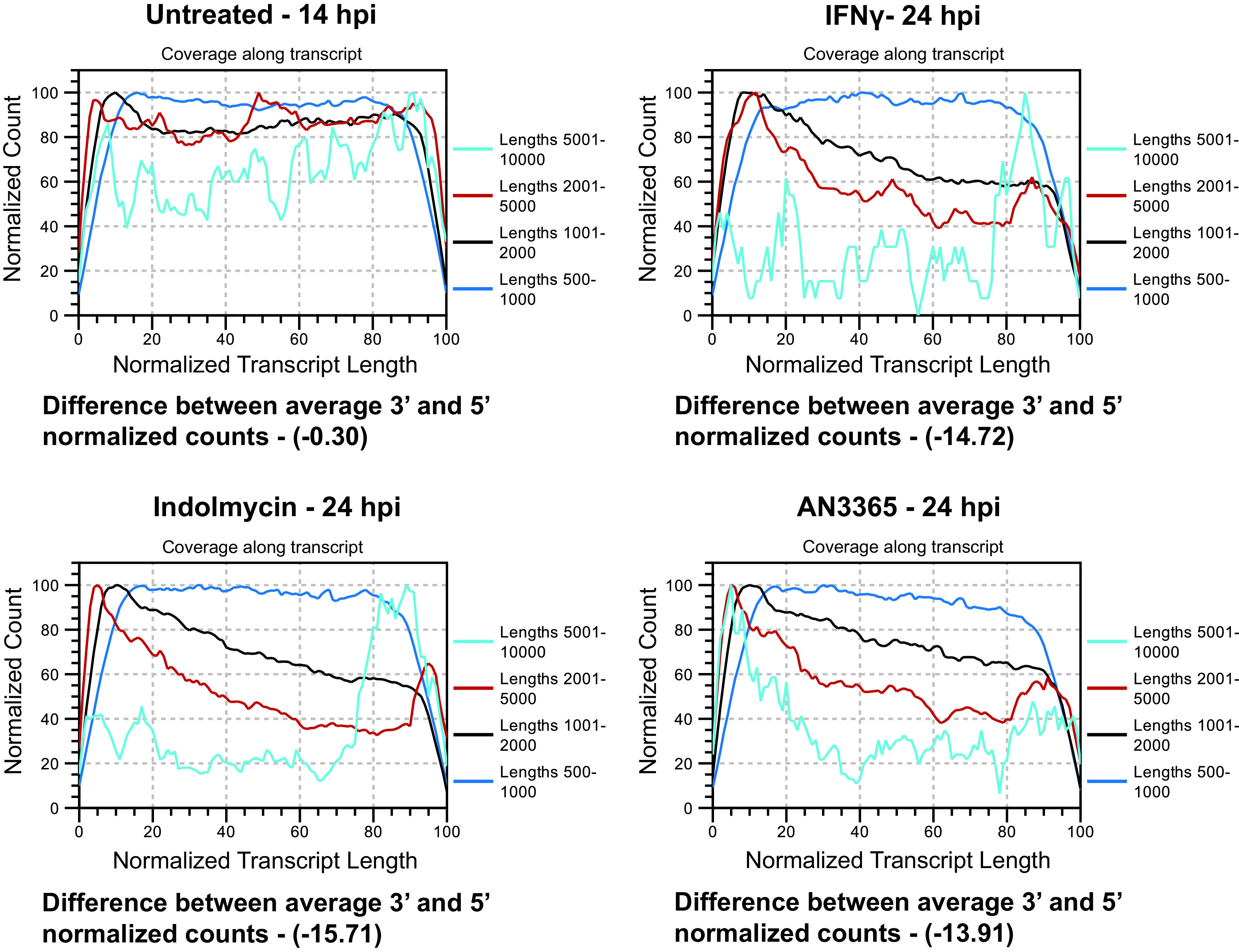
Chlamydial transcript reads of genes larger than 1,000 bp disproportionately map to the 5′ region during persistence. CLC Genomics RNA-seq summary reports provide a broad overview of actual versus expected transcript mapping segregated by gene length. Representative reports are presented for each indicated sample. Below each figure is the difference between average 3′ and 5′ normalized counts of all genes within the sample. A negative number indicates the read counts at the 3′ end are lower than those at the 5′ end for a given gene. Note the general consistency (i.e., horizontal line) across the normalized transcript length (*x* axis) for all conditions for gene sizes between 500 and 1,000 bp (average % Trp = 0.96). In the range 1,000 to 5,000 bp (374 genes; average % Trp = 1.00), there is a clear discrepancy between the mapped 5′ and 3′ regions, suggesting incomplete transcription is occurring in that subset of genes, which is sufficient to skew the average difference between the 3′ and 5′ counts negative. The line 5,001 to 10,000 should not be considered accurate, as only 3 C. trachomatis L2 genes exceed 5,000 bp.

We previously observed that transcript levels at the 5′ and 3′ ends of large genes and operons display discordance, such that the transcript levels at the 3′ ends can be much lower than expected (22). We linked this phenotype to the effect of premature transcript termination mediated by Rho after ribosome stalling during starvation conditions (24). The analysis software for our RNA-seq data sets for the different persistence models revealed this to be a consistent finding. As seen in Fig. 4, larger transcripts showed a skewed ratio of reads between the 5′ and 3′ ends for persistent samples in comparison to the untreated control. This is expected to skew our codon content analyses as transcripts are reported as reads per million base pairs (i.e., TPM [transcripts per million]) such that an increase in transcript reads at the 5′ of an open reading frame (ORF) could be masked by a decrease in transcript reads at the 3′ end. This could conceivably result in Trp codon-rich transcripts displaying no change or even a decrease in transcript levels between time points. To examine this possibility, we mapped transcript reads for different genomic regions directly onto the chromosome to determine whether translational stalling during amino acid starvation would lead to premature transcription termination.

**FIG 5:**
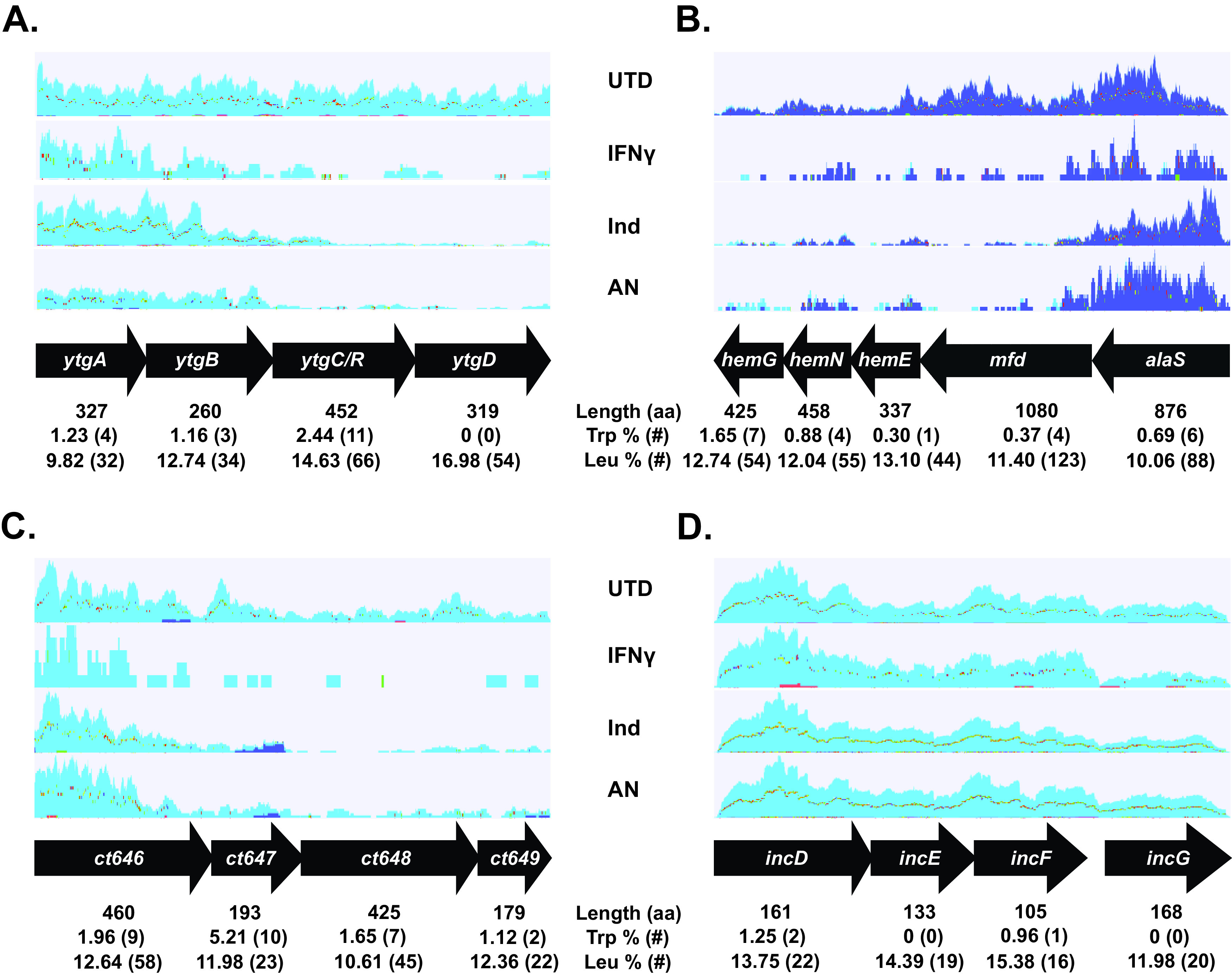
Reads mapped to operons demonstrate correlation between tryptophan (W) content, length, and readthrough efficiency. Transcript reads from a representative replicate of the RNA-seq data were mapped to four operons over four conditions: untreated (UTD) at 14 hpi, IFN-γ treated at 24 hpi, indolmycin (Ind) treated at 24 hpi, and AN3365 (AN) treated at 24 hpi. (A) The *ytg* operon. (B) The *alaS*-to-*hemG* operon containing generally large but Trp codon-poor genes. (C) The *ct646*-to-*ct649* operon containing Trp codon-rich genes. (D) The *incDEFG* operon containing smaller and Trp codon-poor genes. Light blue mapping indicates transcription of the positive strand of the chromosome, while dark blue mapping indicates transcription of the negative strand. The average protein length for C. trachomatis L2 is 353 amino acids (aa), and the average % Trp and % Leu are 0.94 and 11.32, respectively.

We first examined the *ytg* operon, as we have previously demonstrated that its transcription is highly sensitive to Trp limitation (22, 24, 38). A schematic of the operon along with the Trp and Leu content of the encoded proteins is illustrated in Fig. 5A. During the normal developmental cycle, transcripts are detected along the entire operon length, with decreasing levels from the 5′ to 3′ end, as expected (only 14-hpi data are shown for simplicity, with differences between 10- and 24-hpi samples reflecting the greater read depth in the latter sample). During indolmycin treatment, there are no obvious differences between the early treatment and the untreated samples (not shown). However, by 24 hpi (14 h posttreatment), transcripts are not detected in the 3′ genes of the operon, creating a skew between the expected ratios of the 5′ and 3′ ends of the polycistronic message. Both IFN-γ and AN3365 treatments resulted in the rapid loss of 3′ transcripts within the operon, even at 14 hpi (not shown), and this loss of 3′ transcripts persisted for the duration of the analysis. Overall, these data are consistent with our prior results (22, 24, 25) and reinforce that both IFN-γ and AN3365 enact a rapid and potent starvation effect compared to indolmycin, which takes longer to exert its effects.

We next wanted to assess transcript read effects on larger genes that encode proteins with low Trp content. To do this, we mapped transcript reads to the region spanning *alaS* to *hemG*, covering 5 genes total in an apparent operon structure. Again, the Trp and Leu content along with the size of the encoded proteins is illustrated in Fig. 5B. As expected, transcripts decline from the 5′ to 3′ direction under the untreated condition, and no obvious effect of brief indolmycin treatment was observed. Although *alaS* encodes only 6 Trp residues (0.65%), we observed the same patterns as for the *ytg* operon, such that transcripts for the 3′ genes were almost undetectable with longer indolmycin treatment or at any point during IFN-γ and AN3365 treatments. These findings are consistent with our observed effects of downregulated genes being larger than the genome average, suggesting that transcription elongation is negatively impacted during amino acid starvation.

We then mapped transcript reads to the region spanning *ct646* to *ct649*, encoding proteins with a high percentage of Trp (Fig. 5C). Again, we observed the same patterns for mapped transcripts as for the *ytg* operon and the region from *alaS* to *hemG*. Transcripts for *ct646* were detected under all treatment conditions, but transcripts for the 3′ genes were severely attenuated. The 3′ Trp-rich genes display no upregulation by RNA-seq, which goes against the codon effects we observed; however, their transcription is impacted by the Trp codon richness of the 5′ gene.

One trend among the RNA-seq data for all persistent samples was the apparent upregulation of multiple *inc* genes, encoding inclusion membrane proteins that are secreted by the type III secretion system (39, 40). A caveat to these data is that many *inc* genes are also increased between 10 and 14 hpi during the normal developmental cycle (Table S2), suggesting that their increase during persistence may simply reflect an attempt by chlamydiae to continue their developmental cycle program. Nonetheless, we mapped transcripts for the *incDEFG* operon for all conditions to determine whether the same effects we noted above were also observed for these genes (Fig. 5D). Notably, each of the encoded proteins is smaller than the proteome average, Trp poor, and Leu rich. Consistent with the upregulation of these genes as assessed by fold changes over time, we observed no effects on mapped transcript reads in relation to operon position for any treatment condition. Overall, the upregulation of these genes is consistent with the global pattern of smaller genes being overrepresented in the upregulated data sets.

### Trp codon-dependent transcriptional changes during Trp starvation are not mediated by the stringent response in Streptococcus pyogenes.

Chlamydia species are unusual in lacking a stringent response (13). One possible explanation of our data is that these codon-dependent transcriptional changes result from the inability to synthesize ppGpp to modulate transcription: thus, the chlamydial response to amino acid limitation reflects a derepressed state. Alternatively, these codon-dependent changes could reflect an evolved response specific to tryptophan. The simplest means to address this would be to assess transcriptional changes of another tryptophan auxotroph in response to tryptophan starvation in the presence and absence of a stringent response. Streptococcus pyogenes, an important human pathogen, is a natural tryptophan auxotroph for which a *relA* mutant strain has been created (11). Therefore, we cultured both wild-type and *relA* mutant isogenic strains of S. pyogenes in the presence and absence of tryptophan for 1 h, collected RNA, and analyzed transcriptional changes by RNA-seq as a function of the amino acid content of the encoded protein.

**FIG 6:**
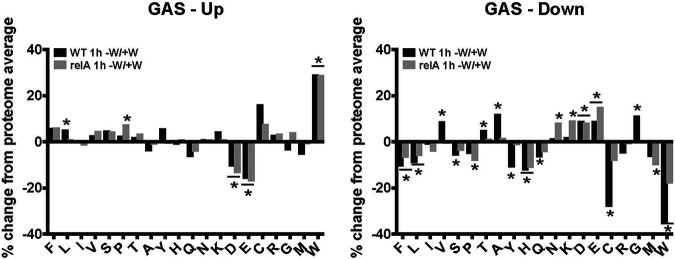
Streptococcus pyogenes transcriptional changes during tryptophan (W) starvation are positively associated with W codon content, regardless of stringent response functionality. Wild-type or *relA* mutant strains of S. pyogenes were cultured in DMEM with (+) or without (−) W for 1 h prior to sample collection. Significant transcripts were defined as >2-fold changed, with a *P* value of <0.05, between the +W and −W conditions for each strain and were calculated based on all biological replicate data. Significant transcripts were graphed in accordance with the relative percentage of change in amino acid codon content compared to the proteome average. A two-tailed, unpaired Student's *t* test assuming equal variance was used to compare each subset of transcripts’ codon content to the proteome average. *, *P* < 0.05; *, both samples are significantly different from the proteome average.

Strikingly, and as seen in Fig. 6, we observed that, like Chlamydia, S. pyogenes also showed an enrichment in Trp codons within the set of genes that were significantly upregulated during tryptophan starvation. The proteome average for each amino acid and protein length, as well as the overall %GC, for S. pyogenes are shown in Table 2 (41). The largest magnitude of change that was statistically significant among the upregulated gene set for both strains was for Trp codon content. This effect was independent of the stringent response, as both the wild-type and *relA* mutant strains displayed this result, with each strain showing a roughly 29% increase in Trp codon content within the upregulated gene set (∼200 ORFs for each). Additional statistically significant codon effects were observed for Asp and Glu, which were less represented within the upregulated gene set. Slight differences between the strains did exist, with the wild type showing an enrichment in Leu codons (5.4%), whereas the mutant showed an enrichment in Pro codons (7.6%). In contrast to Chlamydia, significant effects related to gene length were not observed for Streptococcus, suggesting that this effect is unique to Chlamydia. Notably, Streptococcus does not contain a *rho* ortholog, which encodes a transcription terminator that we have shown can mediate premature transcription termination in Chlamydia during amino acid starvation (24).

Among the genes that were downregulated during Trp starvation, again no meaningful differences were observed between the Streptococcus strains. Trp codons were significantly underrepresented in downregulated transcripts, as were His codons for both strains. Some significant differences were observed between strains, but the trends were similar between them. For example, Cys codons were significantly underrepresented (−28%) in the wild-type strain compared to the mutant (−8.2% [not significant]) in the downregulated gene set (more than 200 ORFs for each). Similarly, Gly codons were significantly enriched (11%) in the wild-type strain compared to the mutant (unchanged) in the downregulated gene set. The biological significance of these other codon effects is not clear. Overall, our transcript analyses of S. pyogenes wild-type and stringent response mutant strains during Trp starvation indicate that the preferential upregulation of Trp codon-containing genes is not unique to Chlamydia and is not dependent on a stringent response.

### Transcript stability during tryptophan starvation in Streptococcus.

We next sought to measure transcript stability in S. pyogenes during Trp starvation to determine whether the increase in Trp codon-containing transcripts could be attributed or not to an increase in stability of these transcripts due to ribosome stalling. As Streptococcus can be grown in pure culture, as opposed to Chlamydia, which requires a host cell in which to propagate, we performed RNA-seq on wild-type cultures starved or not for Trp and treated or not with rifampin for 15 min to block RNA polymerase activity. The half-life of each transcript under the different conditions was then calculated. To focus our analysis on inherently more unstable transcripts, we first filtered our data set for transcripts with half-lives of less than 10 min (HL < 10). This represented 87% of ORFs (*n* = 1,479). We then measured the average half-life of transcripts containing less than 0.6% Trp codons and those containing greater than 1.05% Trp codons (group A streptococcus [GAS] average of 0.83%). These cutoffs were used based on our data from Fig. 6, which showed a roughly 25% increase in the Trp codon content of upregulated transcripts.

Surprisingly, we measured a generalized increase in the stability of GAS transcripts during Trp starvation, similar to what we observed for Chlamydia (Table 6). Under normal growth conditions, the average half-life of transcripts within our filtered data set was 3.05 min, whereas, during Trp starvation, the average half-life of transcripts was 21.2 min (Table 6). Among those transcripts with less than 0.6% Trp codons, the average half-lives under each condition were 2.93 and 20.2 min, whereas for those transcripts with greater than 1.05% Trp codons, the average half-lives were 3.25 and 24.4 min, respectively. Transcripts with higher Trp codon content were statistically significantly more stable under both culture conditions, but there was approximately a 10% increase in stability during Trp starvation. Interestingly, the average size of Trp codon-rich transcripts was smaller than the average size of Trp codon-poor transcripts (895 bp versus 937 bp). Looking only at Trp codon-free transcripts, these displayed half-lives of 3.02 min under normal culture conditions but 19.3 min during Trp starvation. However, these transcripts were also disproportionately smaller than the genome average (640 bp), which complicates interpretation vis-à-vis codon-dependent stability effects.

**TABLE 6:** Streptococcus pyogenes mRNA half-life is not correlated with Trp codon content^*a*^

Category	Culture	mRNA HL (min) with:			
		>1.05% W	<0.6% W	0% W	All
HL < 10 (*n* = 1,478)	+W	3.25 (*n* = 450)	2.93 (*P* < 0.001) (*n* = 710)	3.02 (*P* < 0.025) (*n* = 365)	3.05
	−W	24.4	20.2 (*P* < 0.025)	19.3 (*P* < 0.025)	21.1 (*P* < 0.001)

HL < 3 (*n* = 975)	+W	2.43 (*n* = 275)	2.44 (NS) (*n* = 495)	2.43 (NS) (*n* = 251)	2.44
	−W	14.7	13.5 (NS)	11.5 (*P* < 0.025)	14.0 (*P* < 0.001)

3 < HL < 10 (*n* = 507)	+W	4.54 (*n* = 175)	4.07 (NS) (*n* = 215)	4.32 (NS) (*n* = 114)	4.21
	−W	39.6	35.7 (NS)	36.5 (NS)	34.9 (*P* < 0.001)

a S. pyogenes was cultured in the presence (+) or absence (−) of tryptophan (W) and in the presence or absence of rifampicin (Rif) for 15 min to block RNA polymerase activity. RNA was collected under each condition and analyzed by RNA-seq. The half-lives of transcripts were calculated as described in Table 5 and analyzed based on W codon content. Data are expressed in minutes and assessed for three different categories of HL measurement: <10 min (HL < 10), <3 min (HL < 3), and between 3 and 10 min (3 < HL < 10). The total number of genes in each set is noted by *n* for the HL category and only listed for the +W set within each W culture condition to avoid redundancy. A two-tailed, unpaired Student's *t* test assuming equal variability was used for statistical significance and is indicated for the comparison of the low-% W genes to the high-% W genes, except for the All category, where the comparison is between the +W and −W culture conditions. As a reference, the average W codon contents within all >1.05% and <0.6% categories are >1.75% W and <0.2% W, respectively. See also Table S7 and text for more details. NS, not significant.

10.1128/mSystems.01269-21.8 TABLE S7 Complete RNA-seq analysis of wild-type S. pyogenes NZ131 transcript stability, measured as half-lives (HL), during normal and tryptophan-free (+W and −W, respectively) culture conditions with codon content analysis. Download Table S7, XLSX file, 2.3 MB. Copyright © 2021 Ouellette et al. 2021 Ouellette et al. https://creativecommons.org/licenses/by/4.0/ This content is distributed under the terms of the Creative Commons Attribution 4.0 International license.

Using a more stringent analysis to focus on the most unstable transcripts with half-lives of less than 3 min during normal culture conditions (HL < 3; *n* = 975), there was surprisingly no statistically significant difference between Trp codon-poor and Trp codon-rich transcript stability (+W = 2.44 versus 2.43; −W = 13.5 versus 14.7). This was not true for Trp codon-free transcripts, which displayed decreased stability (+W = 2.42; −W = 11.5) but were again disproportionately smaller than the genome average at 668 bp. The same observations held true for transcripts with half-lives greater than 3 min but less than 10 min (3 < HL < 10; *n* = 503), although there was a not statistically significant trend for Trp codon-rich transcripts to be more stable during Trp starvation (+W = 4.07 versus 4.53; −W = 35.7 versus 39.6). Trp codon-free transcripts displayed no change in this data set (+W = 4.32; −W = 36.5).

An alternative explanation for the data we measured in Fig. 6 is that those genes that displayed an increase in abundance also showed an increase in fold change of stability. To test this, we calculated the fold change increase in stability during Trp starvation and analyzed the Trp content of the 200 most “stable” transcripts (with HL < 10 during normal culture conditions). These possessed an average Trp content of 0.92%, about 10% higher than the proteome average, whereas the 200 most “unstable” transcripts possessed an average Trp content of 0.7%, about 15% lower than the proteome average (see Table S7 in the supplemental material). However, there was limited overlap between these genes and the genes showing the greatest increase or decrease in overall transcript levels (see Fig. S1 in the supplemental material). This indicates that the genes showing the highest fold change in stability were not generally those showing the highest increase in transcript levels during Trp starvation. Overall, we conclude from these transcript stability analyses that the increase in Trp codon content of transcripts we measured during Trp starvation in our bulk RNA-seq experiments cannot be explained solely by ribosome stalling at Trp codons leading to protection of the mRNA transcript.

10.1128/mSystems.01269-21.1 FIG S1 There is no correlation between transcripts with increased stability and those that are increased during tryptophan starvation in Streptococcus. Transcripts showing the highest or lowest fold change (FC) in transcript stability (Stab.) were compared to those transcripts showing the largest or smallest increase during tryptophan starvation (see Table S7). The analysis was performed using Venny 2.1 (J. C. Oliveros, 2007 to 2015, https://bioinfogp.cnb.csic.es/tools/venny/index.html). Download FIG S1, EPS file, 2.1 MB. Copyright © 2021 Ouellette et al. 2021 Ouellette et al. https://creativecommons.org/licenses/by/4.0/ This content is distributed under the terms of the Creative Commons Attribution 4.0 International license.

## DISCUSSION

C. trachomatis is the world’s most common bacterial sexually transmitted infection. In the United States alone, over 1.75 million cases were reported in 2018 (42). This figure is likely a gross underrepresentation given the often asymptomatic nature of a chlamydial infection. These asymptomatic infections, when left untreated, can cause lifelong sequelae, including pelvic inflammatory disease, tubal factor infertility, and ectopic pregnancy (43). In addition, many untreated individuals may remain infected due to Chlamydia’s resistance to natural clearance via the immune system (44). It is hypothesized that the main factor contributing to Chlamydia’s resistance to clearance is its ability to enter persistence (44).

IFN-γ is a known antichlamydial immune effector molecule (8, 18, 21). Its function in immunity to chlamydial infection in mouse models is well established (45–51), and elevated levels of this cytokine have also been detected in infected patient samples (14). In human cell culture, IFN-γ-induced IDO activity, which leads to a Trp-limiting environment, is the key mediator of chlamydial growth inhibition (8, 15). Similarly, IDO depletion of Trp inhibits growth of beta-hemolytic streptococci when cultured with eukaryotic cells, likely due to continued import and degradation of Trp from the extracellular environment (52), thus demonstrating that extracellular bacteria can be inhibited by IFN-γ-mediated IDO production as well (53–55). However, IDO induction is not cidal to pathogenic Chlamydia species, like C. trachomatis and C. pneumoniae, that infect humans. These species are both auxotrophic for Trp, and IDO-mediated depletion of Trp results in persistence. In spite of decades of research investigating chlamydial persistence, there remains a significant gap in our understanding of the molecular mechanisms that drive persistence.

The canonical response to amino acid limitation in model bacterial species is the activation of the stringent response and the production of (p)ppGpp by the ribosome-associated RelA or SpoT (12). This penta/tetraphosphorylated guanosine acts as a signaling molecule to, among other things, alter gene transcription through its effects on RNA polymerase and open complex formation at promoters. In evolving to obligate intracellular dependence, Chlamydia has eliminated the stringent response (56). Thus, what effect an uncharged tRNA binding in the A site of the ribosome has on Chlamydia is not defined.

We recently demonstrated that, in C. pneumoniae, transcriptional changes during IFN-γ-mediated persistence were linked to the Trp codon content of the transcript (22). Given this, we sought to combine multiple persistence models, each acting through amino acid limitation, with RNA sequencing of C. trachomatis to determine whether potential patterns in gene regulation would arise that might highlight a common regulatory pathway that is induced under these conditions. We hypothesized that the codon-dependent transcriptional changes we observed would be (i) specific to the limiting amino acid, (ii) conserved in C. trachomatis, and (iii) independent of the stringent response. To address the last possibility, we determined what role the lack of a stringent response played in chlamydial transcriptional changes by comparing transcriptional changes of wild-type and *relA* mutant strains of S. pyogenes, another Trp auxotroph, to Trp limitation. This would allow us to determine whether the chlamydial response was unique or characteristic of a broader, conserved response to amino acid limitation.

Surprisingly, despite observing changes in chlamydial transcription in each model, these changes were not associated with predictable or logical effects on physiological functions. For example, while some genes in a pathway may be upregulated, other genes within the same pathway may be downregulated. Such was the case for KEGG-annotated genes involved in glycolysis and the tricarboxylic acid (TCA) cycle (see Table S3 in the supplemental material). Thus, our findings suggest that transcriptional profiles during IFN-γ-mediated persistence cannot be relied upon to predict the physiological state of the organism during stress. This is further underscored by the fact that there are relatively few differences between the most transcribed and least transcribed genes across all conditions (using the transcript per million [TPM] data from the RNA-seq experiments). For example, among the 50 most transcribed genes, 23 were common to at least 8 of the 9 conditions we tested (see Table S4 in the supplemental material). Not surprisingly, these genes included those coding for ribosomal proteins, *ompA* (encoding the major outer membrane protein), *tufA* (encoding EF-Tu), *fusA* (encoding EF-G), and *ndk* (encoding nucleoside diphosphate kinase). An additional 8 genes were common to at least 5 of the 6 persistent samples. Among the least transcribed genes, 19 of 50 were common to at least 8 of the 9 conditions we tested. These included a number of hypothetical genes, *fliI* and *flhA* (encoding secretion components), *malQ* (encoding 4-α-glucanotransferase), and *ftsQ* (encoding a cell division protein). Rather, our RNA sequencing data indicate that, during Trp starvation, whether induced by IFN-γ or indolmycin (targeting tryptophanyl-tRNA synthetase), upregulated genes contain significantly more Trp codons than the genome average. This finding is consistent with what we observed for C. pneumoniae (22). Importantly, the Trp codon effect was not observed during AN3365 treatment, which targets leucyl-tRNA synthetase, indicating that there is specificity to the response.

10.1128/mSystems.01269-21.4 TABLE S3 Genes involved in glycolysis are differentially regulated during chlamydial persistence. Download Table S3, DOCX file, 0.02 MB. Copyright © 2021 Ouellette et al. 2021 Ouellette et al. https://creativecommons.org/licenses/by/4.0/ This content is distributed under the terms of the Creative Commons Attribution 4.0 International license.

10.1128/mSystems.01269-21.5 TABLE S4 Comparison of the 50 most highly transcribed C. trachomatis serovar L2 genes under normal and persistent growth conditions as determined by RNA-seq transcript per million (TPM) data. Download Table S4, XLSX file, 0.9 MB. Copyright © 2021 Ouellette et al. 2021 Ouellette et al. https://creativecommons.org/licenses/by/4.0/ This content is distributed under the terms of the Creative Commons Attribution 4.0 International license.

Interestingly, the apparent preference for transcribing Trp codon-rich genes during Trp starvation only extends to the 5′ region/ORF, as early transcript termination disproportionately affects the very same genes (i.e., those rich in Trp). This early termination phenomenon is supported by our previous findings during IFN-γ-induced persistence that indicated ribosomal stalling on Trp codons leads to Rho-dependent transcript termination (24). Additionally, such effects may further mask the codon-dependent transcriptional changes, since transcript levels of Trp codon-rich genes at the 3′ end of an operon with a Trp codon-rich gene at the 5′ end may be negatively impacted. The fact that even large, Trp codon-poor genes show decreases in transcript levels during Trp limitation suggests that transcription elongation may be negatively impacted. One possibility may be that the “dropping off” of RNA polymerase from a large ORF sequence allows reloading of it onto the most active promoters, which may explain why smaller genes and/or those being transcribed before persistence is induced (e.g., early genes like *euo* [57] and *incDEFG* [58]) are preferentially enriched in the transcript data. Alternatively, smaller genes may be “enriched” in the upregulated data sets because Rho is unable to efficiently terminate transcription even after ribosome stalling. For instance, Rho is reported to need ∼100 bp after binding its *rut* site before it can translocate along the nascent message and stop RNA polymerase elongation (59). Further work is required to test these possibilities.

The seemingly passive response of Chlamydia to amino acid starvation may reflect its unique evolutionary niche of having lost the capacity to synthesize (p)ppGpp. Alternatively, its response may act as a biological default that predates the evolution of the stringent response. Our analysis of changes in transcription in S. pyogenes after Trp starvation indicates that it too responds with Trp codon-dependent transcriptional changes. Surprisingly, this response was independent of the ability to synthesize (p)ppGpp, as both the wild-type and *relA* mutant strains exhibited the same Trp codon effect. One possibility for this lack of a canonical stringent response to Trp starvation may be that ribosome stalling at Trp codons is not severe enough to activate RelA. This requires further study. Nevertheless, given the evolutionary divergence between Chlamydia and Streptococcus, our data suggest that their responses to Trp starvation represent an unrecognized mechanism for adapting to limitations of this essential amino acid.

One possible explanation for our findings that Trp codon-rich transcripts are disproportionately enriched during Trp starvation of these Trp auxotrophs is that ribosome stalling at Trp codons leads to protection of the mRNA template. In other experimental systems, ribosome stalling can stabilize a transcript, yet a recent report indicated that mRNA in yeast is cotranslationally degraded (60–62). We previously measured stability of a subset of transcripts in C. pneumoniae during IFN-γ-induced persistence and observed that, while transcripts were generally more stable, there was no Trp codon dependence to this effect (24). We also tested this in C. trachomatis and made the same observations, suggesting that ribosome stalling at Trp codons does not lead to an increase in stability of the associated transcript. However, for Chlamydia, it is challenging to perform a genome-wide assessment of transcript stability during persistence because the biomass—more specifically the mRNA pool—of the organism is already limiting in comparison to the host cell’s, which precludes accurately and quantitatively measuring rifampin-treated mRNA pools. However, these problems do not exist for Streptococcus, which can be grown in pure culture. Therefore, we performed an RNA-seq analysis to determine half-lives of GAS transcripts in the presence and absence of Trp. Surprisingly, we made the same observations as with Chlamydia—transcripts were generally more stable during Trp starvation, but this effect could not be attributed to Trp codon content. Superficially, an increase in transcript stability could be due to a decrease in degradation, but this requires further investigation to determine if RNase expression or activity is negatively impacted by Trp starvation in these organisms. Related to this, a recent report by Hamouche et al. (63) found that rifampin treatment in Escherichia coli leads to the preferential degradation of rRNA transcripts over mRNA transcripts; thus, we cannot formally exclude a similar effect in Chlamydia and Streptococcus that might mask stabilization of a subset of transcripts in a codon-dependent manner. Alternatively, ribosomes could be stalling or slowing along all transcripts, irrespective of their Trp codon content. A similar effect of slowed translation elongation leading to increased transcript stability was recently described in yeast (64). Regardless, for both of these bacteria, our current data indicate an increase in the levels of Trp codon-containing transcripts during Trp starvation cannot be assigned to protection of these specific transcripts from ribosome stalling.

How then does Chlamydia go from sensing a Trp-limiting environment to persistence? Clearly, some Trp-specific responses are enacted, such as the upregulation of the *trpBA* operon that allows for the conversion of indole to Trp (65, 66). Some of the transcriptional changes we observed are likely due to the inability to efficiently translate transcription factors. For example, we and colleagues recently demonstrated that the presence of a triple-Trp motif (WWW) in the YtgCR coding sequence blocks efficient translation of the iron-dependent YtgR repressor (38). Broad translational effects related to Trp have been observed during persistence (67, 68), and it is likely that some predictions could be made about proteins that are more likely to be inefficiently translated (69). In this context both Trp codon density and total Trp codons may be equally important. Some of the most Trp codon-rich genes include the branched-chain tRNA synthetases; thus, one scenario could see Trp starvation leading to the inability to aminoacylate branched-chain amino acid tRNAs, which would then have wide-ranging effects on all protein translation (thus impacting transcription of large genes with no Trp codons, as noted above). Further work is required to test this, yet our data using the leucyl-tRNA synthetase inhibitor AN3365 clearly indicated a rapid and strong effect on chlamydial growth vis-à-vis inducing persistence.

Our analysis indicates that transcriptional profiling during chlamydial persistence, particularly induced by amino acid starvation, must be carefully interpreted. The global effects on transcription related to incomplete transcripts render any efforts at deciphering alterations in specific physiological pathways problematic at best and incomprehensible at worst. That such a response may be conserved across diverse bacteria also highlights the potential for these effects to confound interpretation of other data sets. Overall, our data have revealed a novel, previously undescribed mechanism for Trp starvation responses in Trp auxotrophic bacteria.

## MATERIALS AND METHODS

### Organisms, cell culture, and chemicals.

The human epithelial cell line HEp-2 was routinely cultivated at 37°C with 5% CO_2_ in Dulbecco’s modified Eagle’s medium (DMEM; Gibco, Dun Laoghaire, Ireland) supplemented with 10% fetal bovine serum (FBS). The HEp-2 cells were a kind gift from H. Caldwell (NIH/NIAID). C. trachomatis serovar L2 (434/Bu) EBs were harvested from infected HEp-2 cell cultures at 37°C with 5% CO_2_ and density gradient purified. Titers of purified EBs were determined for infectivity by determining inclusion-forming units (IFU) on fresh cell monolayers. All bacterial and eukaryotic cell stocks were confirmed to be *Mycoplasma* negative using the LookOut *Mycoplasma* PCR detection kit (Sigma, St. Louis, MO). S. pyogenes strain NZ131 and a *relA* mutant derivative (NZ131 *relA*::pRF2) were obtained from Host Malke and have been previously described and characterized (11). The mutant consists of a *relA* gene truncated at codon 129 due to the insertion of a gene conferring resistance to erythromycin.

Indolmycin was purchased from Cayman Chemical (Ann Arbor, MI) and resuspended to 120 mM in dimethyl sulfoxide (DMSO; Sigma). Aliquots were kept at −80°C and used only once to avoid freeze-thawing. Indolmycin was used at 120 μM and added at 10 hpi in all C. trachomatis experiments. Immediately prior to treatment, cell medium was replaced with DMEM lacking Trp (made in-house) to enhance the inhibitory effects of indolmycin (see reference 25 for more information). DMEM lacking Trp was made using 10% fetal bovine serum that had been dialyzed to remove any additional amino acids. All custom medium components and any other chemicals were purchased from Sigma unless otherwise noted.

AN3365 was purchased from Cayman Chemical and resuspended to 5 mg ml^−1^ in DMSO. Aliquots were kept at −20°C and allowed one additional freeze-thaw. AN3365 concentration was titrated to induce persistence without completely stalling development and was used at 1 μg ml^−1^ with treatment at 10 hpi in all C. trachomatis experiments. No modifications to DMEM were necessary.

Recombinant human interferon gamma (IFN-γ) was purchased from Cell Sciences (Canton, MA) and resuspended to 100 μg ml^−1^ in 0.1% bovine serum albumin (BSA; Sigma) diluted in water. Aliquots were frozen at −80°C and used only once to avoid freeze-thawing. IFN-γ was titrated for its effect to induce persistence without killing the bacteria, and in our experiments, 0.5 ng ml^−1^ was added to cells approximately 11 h prior to infection. Medium was replaced at 10 hpi with IFN-γ-conditioned medium (ICM) to induce persistence in C. trachomatis, as described previously (24). ICM was prepared by adding 2 ng ml^−1^ IFN-γ to uninfected HEp-2 cells for approximately 54 h prior to collection and filtration of the medium.

### Nucleic acid extraction and enrichment from Chlamydia.

HEp-2 cells plated in 6-well plates at a density of 10^6^ per well were infected with C. trachomatis serovar L2 at a multiplicity of infection (MOI) of 1. Infected cells were treated or not as described above to induce persistence. At the indicated times postinfection, C. trachomatis RNA extraction was performed on infected cell monolayers using TRIzol according to the manufacturer’s instructions (Invitrogen/Thermo Fisher). Samples were treated with Turbo DNAfree (Ambion/Thermo Fisher) according to the manufacturer’s instructions to remove DNA contamination. A 1-μg aliquot of RNA was set aside for reverse transcription-quantitative PCR (RT-qPCR) as a preenrichment control. The remaining RNA was treated using Dynabeads (Ambion/Thermo Fisher) to remove host mRNA contamination, with slight amendments to the manufacturer’s instructions—notably, the supernatant was saved for further processing and the bead-bound mRNA was discarded. Samples were then treated using MICROB*Enrich* and MICROB*Express* (Invitrogen/Thermo Fisher) according to the manufacturer’s instructions. Samples were aliquoted for RT-qPCR, BioAnalyzer analysis, and cDNA library preparation. Three biological replicates were collected.

### Isolation, processing, and analysis of RNA from tryptophan-starved S. pyogenes.

Briefly, either the wild-type or *relA* mutant strain of S. pyogenes NZ131 was used to inoculate amino-acid-free Dulbecco’s modified Eagle’s medium (DMEM) F-12 with or without tryptophan supplemented with glucose (4.65 g/liter), 5% casein hydrolysate (CAA), 0.5 mg ml^−1^ sodium thioglycolate (STG), and 0.1 g liter^−1^ exogenous l-tryptophan. Of note, 2 μg ml^−1^ of erythromycin was included in *relA* mutant cultures. Cultures were incubated at 37°C in 5% CO_2_ overnight, and then the bacteria were pelleted, suspended in phosphate-buffered saline (PBS), and diluted to an optical density at 600 nm (OD_600_) of 0.1 in DMEM F-12 (plus CAA, STG, and Trp) and incubated at 37°C in 5% CO_2_ for 4 h. Following incubation, bacteria were pelleted and rinsed with tryptophan-free DMEM F-12 (plus CAA and STG) three times to remove tryptophan. Bacteria were suspended with DMEM either containing or lacking exogenous l-tryptophan and incubated at 37°C in 5% CO_2_. Ten-milliliter samples were taken 1 h after suspension in medium with or without tryptophan. The bacteria in the samples were pelleted, rinsed with PBS, suspended in RNAlater (Qiagen), and frozen at −80°C. Dilution plating of samples obtained at the time of suspension in medium containing Trp or not, as well as 1 and 4 h later, was done to determine CFU/ml. To extract and purify total RNA, frozen bacterial cells were pelleted and lysed using a FastRNA Pro Blue kit (MP Biomedicals) with 2 agitation cycles of 40 s at a speed of 6 with a FastPrep homogenizer (Thermo Scientific). The supernatant was then processed using the RNeasy minikit (Qiagen). Ten micrograms of the resulting RNA was treated with DNase using the Turbo DNA-Free kit (Invitrogen) prior to use in qRT-PCR and RNA-seq. Three biological replicates were performed.

### Library preparation and RNA sequencing.

Beginning with 300 ng of total RNA from the sample, RNA-seq libraries were prepared using the TruSeq stranded total RNA library prep (Illumina, Inc. San Diego, CA) following the recommended protocol with the following modification for Chlamydia. In the step in which the total RNA is incubated with Ribozero to remove ribosomal transcripts, the RNA is incubated with 5 μl total solution consisting of a combination of 2.5 μl human/mouse/rat (HMR) Ribozero solution and 2.5 μl of bacterial Ribozero (Illumina). This facilitates the removal of ribosomal transcripts of both the host and the bacterial components of the RNA mixture. For Streptococcus, 5 μl of bacterial Ribozero solution was used, since these cultures do not have contaminating eukaryotic RNA. The resultant libraries from the individual samples were multiplexed and subjected to 75-bp paired-read sequencing to generate approximately 60 million pairs of reads per sample using a high-output 150-cycle flow cell on an Illumina NextSeq500 sequencer in the UNMC Genomics Core facility.

The original fastq format reads were trimmed by the fqtrim tool (https://ccb.jhu.edu/software/fqtrim) to remove adapters, terminal unknown bases (N’s), and low-quality 3′ regions (Phred score of <30). The trimmed fastq files were processed by FastQC (70). Chlamydia trachomatis 434/Bu and Streptococcus pyogenes NZ131 bacterial reference genomes and annotation files were downloaded from Ensembl (http://bacteria.ensembl.org/Chlamydia_trachomatis_434_bu_gca_000068585/Info/Index or http://bacteria.ensembl.org/Streptococcus_pyogenes_nz131_gca_000018125/Info/Index). The trimmed fastq files were mapped to Chlamydia trachomatis 434/Bu or Streptococcus pyogenes NZ131 by CLC Genomics Workbench 12 for RNA-seq analyses. For C. trachomatis, a total of 9 samples per experiment were analyzed at a time, and three biological experiments were performed. Of the 27 total samples, two failed our quality controls (one 14-h IFN-γ sample and one 10-h plus 4-h indolmycin sample from separate experiments, meaning each of these samples had only two biological replicates). For S. pyogenes, a total of 4 samples per experiment were analyzed, and three biological experiments were performed. Of the 12 total samples, one failed our quality control (one WT plus Trp sample, meaning this sample had only two biological replicates).

### RT-qPCR.

cDNA was synthesized from DNA-free RNA using random nonamers (New England BioLabs, Ipswich, MA) and SuperScript III RT (Invitrogen/Thermo Fisher) per the manufacturer’s instructions. Reaction end products were diluted 10-fold with molecular biology-grade water, aliquoted for later use, and stored at −80°C. Equal volumes of each reaction mixture were used in 25-μl qPCR mixtures with SYBR green master mix (Applied Biosystems) and quantified on a QuantStudio 3 (Applied Biosystems/Thermo Fisher) using the standard amplification cycle with a melting curve analysis. Results were compared to a standard curve generated against purified C. trachomatis L2 genomic DNA. DNA samples were collected from replicate wells during the same experiments, using the DNeasy blood and tissue kit (Qiagen, Hilden, Germany). Equal total DNA quantities were used in qPCR with a *groEL1* primer set to quantify chlamydial genomes. Genome values were used to normalize the respective transcript data. RT-qPCR results were normalized for efficiency, with typical results demonstrating an *r*^2^ value of >0.995 and efficiencies greater than 90%. Student's *t* test was used to compare each 24-hpi value to 10-hpi untreated in C. trachomatis following log_10_ transformation (where * indicates a *P* value of <0.05).

### Immunofluorescence assay.

Cells were cultured on glass coverslips in 24-well tissue culture plates and infected with C. trachomatis at a multiplicity of infection (MOI) of 1. All cells were fixed in 100% methanol. Organisms were stained using a primary goat antibody specific to C. trachomatis major outer membrane protein (MOMP) and a donkey anti-goat secondary antibody conjugated to Alexa Fluor 488 (Jackson Laboratory, Bar Harbor, ME.). DAPI (4′,6-diamidino-2-phenylindole) was added to visualize DNA. Images were acquired on a Zeiss AxioImager.Z2 equipped with an Apotome2 using a 100× lens objective.

### Half-life determination.

mRNA half-life (*t*_1/2_) was calculated using the formula *t*_1/2_ = *t*/{log_2_(*N*_0_) − log_2_[*N*(*t*)]}, where *t* represents the 15 min of treatment with rifampin (Rif), *N*_0_ is the amount of transcript measured at the time of addition of Rif, and *N*(*t*) is the amount of transcript measured after 15 min of Rif treatment. For chlamydial samples, cells were infected and treated or not as described above to induce persistence, and cultures were subsequently treated or not with 1 μg ml^−1^ Rif (diluted from a 1-mg ml^−1^ stock in 100% ethanol) for 15 min. Total RNA samples were collected before and after Rif addition and processed for RT-qPCR as described above. C. trachomatis half-lives were calculated using ng cDNA values. S. pyogenes was grown with 40 ml DMEM F-12 medium with or without tryptophan to the exponential phase of growth. The cultures were centrifuged, and bacterial pellets were rinsed three times with DMEM F-12 medium lacking tryptophan. Prior to the final wash, the bacteria were separated into four 10-ml cultures and centrifuged. Two pellets were suspended with medium containing tryptophan and Rif (250 μg ml^−1^) or not and two with medium lacking tryptophan and containing rifampin or not. After 15 min of incubation at 37°C, the cultures were centrifuged to pellet the bacteria, and RNA was isolated as previously described. Three independent experiments were performed and the 12 samples were processed for RNA-seq. Due to the discontinuation of the rRNA depletion kit used in the processing of samples for our bulk RNA-seq analysis during amino acid starvation, the samples for half-life determination were prepared for RNA-seq as follows. Libraries were generated using 100 ng of total RNA from each sample and using the Universal Prokaryotic RNA-Seq AnyDeplete kit from NuGen (Tecan Genomics, Redwood City, CA) following the recommended procedure. Libraries were multiplexed and sequenced on the NextSeq550 DNA analyzer (Illumina) to generate approximately 12 million pairs of 75-bp reads for each sample. FASTQ files were provided to the Bioinformatics and Systems Biology core for further analysis. S. pyogenes half-lives were calculated using total gene reads provided by RNA-seq, but similar results on transcript stability and Trp codon content were obtained using TPM values, which normalizes based on gene length (see Table S7 for the full data set). Any gene that showed a negative half-life (meaning increased reads) or a value greater than 120 min (i.e., greater than ∼40-fold the average half-life) was arbitrarily set to 120 min to avoid skewing the analysis with biologically meaningless values. This affected 80 ORFs (<0.1% of total ORFs).

### Statistical analyses.

Each gene’s read counts were modeled by a separate Generalized Linear Model (GLM), assuming that the read counts follow a negative binomial distribution, and were normalized based on transcripts per million (TPM). The Wald test was used for statistical analysis of the two-group comparisons. The false-discovery rate (FDR) and Bonferroni adjusted *P* values were also provided to adjust for any multiple-testing problem. Fold changes are calculated from the GLM, which corrects for differences in library size between the samples and the effects of confounding factors.

For the codon analysis, each comparison was rank ordered in Microsoft Excel. For the quintile analysis, each ∼175 genes (i.e., from 1 to 175, 176 to 350, etc.) were analyzed for their average gene length and amino acid content. A Venn diagram was used to represent these data in Fig. 2. For the stringent analysis, all genes showing a greater than 2-fold change in transcript levels with *P* < 0.05 were analyzed for their average length and amino acid content. These data were graphed in GraphPad and shown in Fig. 3. The complete data analysis for Chlamydia can be found in Table S5 in the supplemental material. For data shown in Fig. 6 for S. pyogenes, the complete data analyses can be found in Tables S6 and S7 in the supplemental material.

10.1128/mSystems.01269-21.6 TABLE S5 Complete RNA-seq fold-change analysis of C. trachomatis serovar L2 transcripts during normal and persistent growth conditions with statistical assessment and codon content analysis. U, untreated/normal; Ind, indolmycin; AN, AN3365; IFN, interferon gamma. 10, 14, and 24 refer to the hours postinfection time point comparison. Download Table S5, XLSX file, 0.7 MB. Copyright © 2021 Ouellette et al. 2021 Ouellette et al. https://creativecommons.org/licenses/by/4.0/ This content is distributed under the terms of the Creative Commons Attribution 4.0 International license.

10.1128/mSystems.01269-21.7 TABLE S6 Complete RNA-seq fold change analysis of wild-type (WT) and stringent response mutant (*relA* mutant) strains of S. pyogenes NZ131 transcripts during normal and tryptophan-free (+W and −W, respectively) growth conditions with codon content analysis. Red highlighted values were statistically significant. Download Table S6, XLSX file, 0.7 MB. Copyright © 2021 Ouellette et al. 2021 Ouellette et al. https://creativecommons.org/licenses/by/4.0/ This content is distributed under the terms of the Creative Commons Attribution 4.0 International license.

### Data availability.

The raw and processed RNA sequencing reads in fastq format have been deposited in the Gene Expression Omnibus (GEO; www.ncbi.nlm.nih.gov/geo/) under accession no. GSE174036.
