# Supplementary material for: Codon-Dependent Transcriptional Changes in Response to Tryptophan Limitation in the Tryptophan Auxotrophic Pathogens Chlamydia trachomatis and Streptococcus pyogenes
Source: mSystems. 2021 Dec 14;6(6):e01269-21. doi: 10.1128/mSystems.01269-21 (PMC8670374; doi:10.1128/mSystems.01269-21)
Supplement: TABLE S3 [file msystems.01269-21-st003.docx]

| Name | Gene ID | Ctr D ORF | Gene length | 14U/10U | 24U/14U | 14IFN/10U | 24IFN/14IFN | 24IFN/10U | 14Ind/10U | 24Ind/14Ind | 24Ind/10U | 14AN/10U | 24AN/14AN | 24AN/10U | W | %W |
| --- | --- | --- | --- | --- | --- | --- | --- | --- | --- | --- | --- | --- | --- | --- | --- | --- |
| pgk | CTL0062 | CT693 | 1212 | -1.95 | 4.70 | -1.83 | 1.35 | -1.36 | -1.67 | 3.53 | 2.50 | -1.40 | 5.71 | 4.11 | 2 | 0.50 |
| pckA | CTL0079 | CT710 | 1800 | 1.27 | 1.77 | 1.60 | 1.68 | 2.23 | 1.20 | -1.42 | -1.22 | -1.05 | -1.28 | -1.47 | 18 | 3.01 |
| pgmA | CTL0091 | CT722 | 681 | 1.96 | 1.56 | 1.49 | 1.19 | 1.56 | 1.36 | -1.05 | 1.24 | -1.39 | 1.10 | -1.39 | 4 | 1.77 |
| pfkA | CTL0457 | CT205 | 1662 | 1.47 | 2.14 | -1.37 | -1.49 | -2.22 | 1.24 | -1.81 | -1.54 | -1.57 | 1.12 | -1.26 | 3 | 0.54 |
| pfkA_2 | CTL0459 | CT207 | 1647 | -1.80 | 1.84 | -1.54 | -2.33 | -3.35 | -1.37 | 1.03 | -1.03 | -1.58 | 3.34 | 2.37 | 3 | 0.55 |
| dhnA | CTL0467 | CT215 | 1047 | -1.01 | 1.33 | -1.07 | 1.13 | -1.10 | 1.06 | -1.51 | -1.44 | 1.22 | -1.64 | -1.57 | 1 | 0.29 |
| pdhA | CTL0497 | CT245 | 1023 | -1.05 | 1.16 | 1.45 | -1.52 | -1.15 | 1.12 | 1.16 | 1.27 | 1.11 | 1.00 | 1.01 | 3 | 0.88 |
| pdhB | CTL0498 | CT246 | 987 | 1.57 | 1.01 | 1.08 | -1.33 | -1.35 | 1.19 | -2.06 | -1.77 | -1.60 | -1.04 | -1.85 | 3 | 0.91 |
| pdhC | CTL0499 | CT247 | 1290 | 1.66 | 1.05 | 1.10 | -2.09 | -2.26 | 1.45 | -5.99 | -4.79 | -1.31 | -3.96 | -6.50 | 2 | 0.47 |
| tpiS | CTL0582 | CT328 | 825 | 1.22 | 1.23 | -3.07 | -1.23 | -3.32 | -1.26 | -5.49 | -6.99 | -2.14 | 1.18 | -1.94 | 3 | 1.09 |
| pykF | CTL0586 | CT332 | 1458 | -1.05 | 1.10 | -1.19 | 1.41 | 1.18 | -1.15 | -1.21 | -1.20 | 1.15 | -1.13 | -1.04 | 2 | 0.41 |
| CTL0663 | CTL0663 | CT406 | 465 | 1.09 | -1.08 | -1.09 | -1.06 | -1.14 | -1.01 | -1.24 | -1.08 | 2.27 | -1.57 | 1.66 | 0 | 0.00 |
| gapA | CTL0767 | CT505 | 1005 | 1.30 | -1.09 | 1.19 | 1.10 | 1.22 | 1.31 | 1.58 | 2.04 | 1.10 | 1.60 | 1.68 | 3 | 0.90 |
| lpdA | CTL0820 | CT557 | 1398 | 1.15 | 1.45 | 1.28 | -1.04 | 1.13 | 1.06 | 1.21 | 1.27 | -1.27 | -2.08 | -2.70 | 2 | 0.43 |
| eno | CTL0850 | CT587 | 1275 | 1.43 | -1.02 | 1.83 | -1.13 | 1.42 | 1.28 | -1.17 | 1.19 | 1.66 | -3.34 | -2.12 | 1 | 0.24 |

Supplemental Table 3. Genes involved in glycolysis are differentially regulated during chlamydial persistence.

Supplemental Table 3. Genes involved in the TCA cycle are differentially regulated during chlamydial persistence.

| **Name** | **Gene ID** | **Ctr D ORF** | **Gene length** | **14U/10U** | **24U/14U** | **14IFN/10U** | **24IFN/14IFN** | **24IFN/10U** | **14Ind/10U** | **24Ind/14Ind** | **24Ind/10U** | **14AN/10U** | **24AN/14AN** | **24AN/10U** | **W** | **%W** |
| --- | --- | --- | --- | --- | --- | --- | --- | --- | --- | --- | --- | --- | --- | --- | --- | --- |
| pckA | CTL0079 | CT710 | 1800 | 1.27 | 1.77 | 1.60 | 1.68 | 2.23 | 1.20 | -1.42 | -1.22 | -1.05 | -1.28 | -1.47 | 18 | 3.01 |
| sucC | CTL0193 | CT821 | 1161 | 1.26 | 20.81 | 2.96 | -3.68 | -1.39 | 1.57 | 1.09 | 1.69 | 1.25 | -1.22 | -1.08 | 2 | 0.52 |
| sucD | CTL0194 | CT822 | 876 | 1.69 | 9.76 | 1.63 | 2.61 | 4.27 | 1.27 | 1.11 | 1.57 | 2.48 | -1.18 | 2.22 | 2 | 0.69 |
| sucA | CTL0310 | CT054 | 2712 | 1.46 | 9.12 | -1.51 | 3.34 | 2.13 | 1.14 | 1.18 | 1.42 | 1.67 | 1.24 | 2.06 | 10 | 1.11 |
| sucB_1 | CTL0311 | CT055 | 1098 | 3.11 | 14.62 | 3.94 | -1.63 | 2.12 | 2.05 | -1.04 | 1.97 | 4.18 | -3.34 | 1.08 | 1 | 0.27 |
| pdhA | CTL0497 | CT245 | 1023 | -1.05 | 1.16 | 1.45 | -1.52 | -1.15 | 1.12 | 1.16 | 1.27 | 1.11 | 1.00 | 1.01 | 3 | 0.88 |
| pdhB | CTL0498 | CT246 | 987 | 1.57 | 1.01 | 1.08 | -1.33 | -1.35 | 1.19 | -2.06 | -1.77 | -1.60 | -1.04 | -1.85 | 3 | 0.91 |
| pdhC | CTL0499 | CT247 | 1290 | 1.66 | 1.05 | 1.10 | -2.09 | -2.26 | 1.45 | -5.99 | -4.79 | -1.31 | -3.96 | -6.50 | 2 | 0.47 |
| mdhC | CTL0630 | CT376 | 981 | 1.49 | 1.24 | 1.62 | 1.01 | 1.51 | 1.51 | 1.77 | 2.78 | 2.15 | -1.73 | 1.19 | 5 | 1.53 |
| sucB_2 | CTL0657 | CT400 | 1167 | -1.01 | -1.00 | -1.58 | -1.01 | -1.71 | -1.37 | 1.86 | 1.24 | -1.02 | 2.30 | 2.04 | 2 | 0.52 |
| lpdA | CTL0820 | CT557 | 1398 | 1.15 | 1.45 | 1.28 | -1.04 | 1.13 | 1.06 | 1.21 | 1.27 | -1.27 | -2.08 | -2.70 | 2 | 0.43 |
| sdhB | CTL0854 | CT591 | 699 | 1.49 | 4.03 | -1.48 | -1.80 | -2.46 | -1.05 | -1.00 | 1.08 | -1.45 | 1.38 | 1.02 | 2 | 0.86 |
| sdhA | CTL0855 | CT592 | 1881 | -1.32 | 2.19 | -1.20 | -1.19 | -1.65 | -1.56 | 1.13 | -1.39 | -1.54 | -1.79 | -2.88 | 6 | 0.96 |
